# ROASMI: accelerating small molecule identification by repurposing retention data

**DOI:** 10.1186/s13321-025-00968-8

**Published:** 2025-02-14

**Authors:** Fang-Yuan Sun, Ying-Hao Yin, Hui-Jun Liu, Lu-Na Shen, Xiu-Lin Kang, Gui-Zhong Xin, Li-Fang Liu, Jia-Yi Zheng

**Affiliations:** 1 State Key Laboratory of Natural Medicines, Department of Chinese Medicines Analysis, School of Traditional Chinese Pharmacy, China Pharmaceutical University, No. 24 Tongjia Lane, Nanjing, 210009 China; 2 Shenzhen Key Laboratory of Hospital Chinese Medicine Preparation, Shenzhen Traditional Chinese Medicine Hospital, The Fourth Clinical Medical College of Guangzhou University of Chinese Medicine, Shenzhen, 518033 China

**Keywords:** Metabolomics, Retention order, Small-molecule identification, Replicability, Deep learning

## Abstract

The limited replicability of retention data hinders its application in untargeted metabolomics for small molecule identification. While retention order models hold promise in addressing this issue, their predictive reliability is limited by uncertain generalizability. Here, we present the ROASMI model, which enables reliable prediction of retention order within a well-defined application domain by coupling data-driven molecular representation and mechanistic insights. The generalizability of ROASMI is proven by 71 independent reversed-phase liquid chromatography (RPLC) datasets. The application of ROASMI to four real-world datasets demonstrates its advantages in distinguishing coexisting isomers with similar fragmentation patterns and in annotating detection peaks without informative spectra. ROASMI is flexible enough to be retrained with user-defined reference sets and is compatible with other MS/MS scorers, making further improvements in small-molecule identification.

**Supplementary Information:**

The online version contains supplementary material available at 10.1186/s13321-025-00968-8.

## Scientific Contribution

Our work discovers the dependence of buffer pH on the replicability of retention sequences in RPLC systems. Building upon this mechanistic insight, we have constructed a generalizability-oriented retention order prediction model called ROASMI, which is capable of providing reliable predictions across heterogeneous datasets with diverse chromatographic and chemical spaces.

**Supplementary Information:**

The online version contains supplementary material available at 10.1186/s13321-025-00968-8.

## Introduction

Liquid chromatography-mass spectrometry (LC–MS) has greatly facilitated the identification of small molecules of biological interest. Generally, a single run can detect thousands of peaks, including their retention times (RTs), high-resolution m/z values, and, if available, tandem MS (MS/MS) spectra. To extract meaningful structural information from these data, numerous in silico tools based on accurate mass searches or MS/MS spectral analysis have been developed [1–4]. However, there has been little progress in developing in silico methods to effectively utilize retention data, meaning that approximately two-thirds of the detected peaks whose lack usable MS/MS spectra remain to be under-annotated [5–9].

**Fig. 1:**
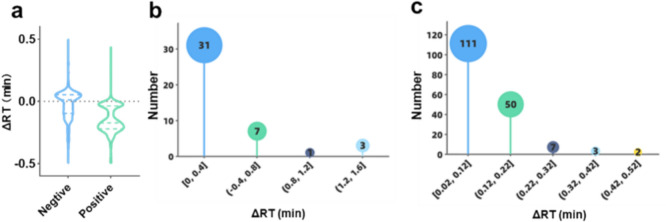
Illustration of the poor replicability of RT based on public metabolomics experiments. The distribution of differences in experimental RTs observed between different configurations (source data: *ST001683* dataset [10]), the difference (△RT) of measurements made with two different types of mass spectrometers under exactly the same experimental conditions, with n = 499 for positive ion mode, n = 631 for negative ion mode (**a**); different detection polarities (source data: *dataset_56* [11]), of which a total of 42 compounds were measured under both negative and positive ion modes, respectively (**b**); and different analytical runs (source data: *dataset_61* [12]), of which a total of 173 compounds were measured in ten replicate runs (**c**)

The underutilization of retention data in automated annotation tools is due primarily to the poor replicability of RTs across different chromatographic methods (CMs). The concept of “replicability”, defined by the National Academies of Sciences [13], refers to consistent retention measurements across studies employing distinct CMs. Achieving replicable RTs poses challenges due to significant variations, even within the same CM (see Fig. 1). Owing to the assumption that the retention order of analytes is more replicable than their RTs are, two types of retention models have been developed. One type involves retention time projection methods, which establish monotonically constrained and nonlinear mappings between the RTs of different datasets [14–18]. The ability to predict RTs for analytes depends on the chemical space similarity (ChemSS) and chromatographic space similarity (ChrSS), which must be estimated separately for each pair of datasets. The other type involves retention order models, which have proven trainable via heterogeneous datasets and applicable across CMs [19–23]. Nonetheless, determining the degree of ChrSS required for replicable retention orders remains to be explored.

Here, we introduce the retention-order-assisted small-molecule identification (ROASMI) model, which has strong generalizability for predicting retention properties across diverse chemical and chromatographic spaces. By leveraging neural network-based molecular representations, ROASMI exhibits accurate prediction capabilities for analytes, even those with different chemical structural similarities to the training set. Additionally, by incorporating mechanistic insights, the model is directly applied to RPLC datasets without projection. To illustrate the applicability of ROASMI in assisting small molecule identification, ROASMI is used independently or in combination with MS/MS tools to distinguish isomers and improve top-k accuracy. More importantly, our work fundamentally revolutionizes how to utilize retention data buried in untargeted metabolomics studies and expands the power of retention information in automated compound annotation.

## Methods

### Collection and curation of RT datasets

In this study, the primary data source was the RepoRT database, which represents the most extensive public repository of RT data available [24]. Meanwhile, supplementary retention data were gathered from the scientific literature and additional databases, including PredRet [15], Metabolome Workbench [25], and MetaboLights [26]. The collected retention data encompassed both metadata and data-level information [27]. Metadata information included details about the experimental methodologies employed in the datasets, such as mobile phase type, buffer pH, column chemistry and geometry, column temperature, gradient, flow rate, instrument specifications, sample type, and DOI number. For the data-level information, we focused on compounds that contain any combination of C, H, N, O, P, and S. The Compound Identifier (CID) numbers for each compound were retrieved from PubChem as of September 1, 2024. The retention datasets were systematically organized and sequentially numbered according to the number of compounds they contain, ranging from *dataset_01* (33 compounds) to *dataset_87* (50,447 compounds).

### Classification and utilization of datasets

The collated datasets were further classified into three categories for different scenarios:

#### Initial reference set

This dataset is used for the initial training of ROASMI and characterized by: (i) a large data size to support the training of deep learning models; (ii) a typical pH value of the buffer solution to ensure better generalizability of the learned retention information. *Dataset_87* fits both which contians 50,447 RT records and a widely used pH value of 2.68 [28].

#### Retraining reference sets

This refers to datasets with similar chemical or chromatographic space as the prediction set. These datasets can come from our collected datasets or can be obtained by users searching the literature and metabolic repositories based on the conditions of the prediction set. The ChemSS (%) between the retraining reference set and the prediction set is determined by dividing the number of overlapping analytes between the two sets by the total number of analytes in the prediction set. ChrSS is scored on the basis of buffer pH, column chemistry, type of organic solvent, column temperature, and other parameters. A score of 1 is given for each category when the retraining reference set matches the prediction set; otherwise, the score is 0.

#### Application sets

Four datasets of different sample types were used to assess the applicability of ROASMI for small-molecule identification: (i) *Dataset_71* [29], which contained samples from human metabolites, was used to evaluate ROASMI's performance in terms of isomer annotation. The original study used 16 representative compounds to establish a multiple linear regression model with two molecular descriptors as inputs, which could be used to distinguish isomer pairs or triplets; (ii) *Dataset_79* [10], which contained samples from gut microbes and many isomer subgroups, such as 14 isomer pairs, four triplets, two quadruplets, and one quintuplet; (iii) the Critical Assessment of Small Molecule Identification (CASMI) 2022 category-4 priority challenge [30], with unknown samples. The raw data were available at https://drive.google.com/drive/folders/1h3Hf8vxDQUhDN9aIRKIOBKGR91qgg3cL?usp=sharing; and (iv) *Dataset_38*, which comprised pooled samples from plants, along with 58 standards [31]. The raw data ('20100917_03_Mixed.mzML') were available from MetaboLights under the identification number MTBLS36. This dataset supported de novo identification and illustrated the application of ROASMI in plant-derived samples, which had fewer MS/MS reference spectra compared to those derived from humans.

#### Retention replicability analysis

Using the correlation coefficient of retention sequences (CCRS) as an indicator, the influences of pH, column chemistry, column temperature, and the type of organic solvent on retention replicability were separately examined. Here, the similarity of column chemistry was assessed based on the USP-PQRI procedure [32], which assumes that the similarity between two columns can be represented by a quantity F with a smaller F value indicating higher similarity. Using the Zorbax Extend C18 column as a baseline with a default pH of 2.8, F values for each column were obtained from the PQRI database (https://apps.usp.org/app/USPNF/columnsDB.html). If a column was not in the PQRI database, its F value was expressed as 'n/a'.

#### The architecture of ROASMI

**Fig. 2:**
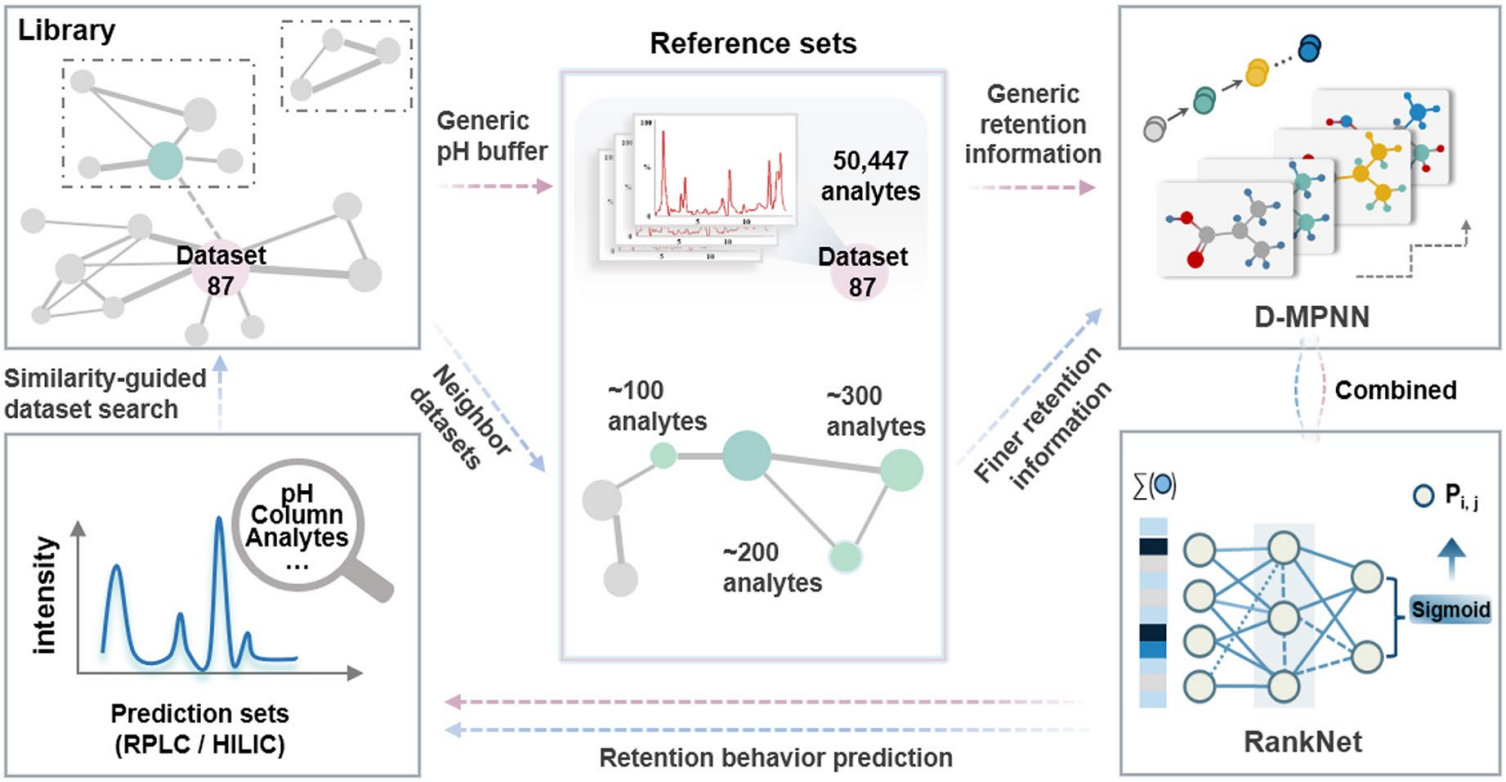
Overview of the training process for the ROASMI model. The model can be trained in two phases: an initial training phase (indicated by the red line) and a retraining phase (indicated by the blue line). In the initial training phase, *dataset_87* is used as an initial reference set to train D-MPNN to learn generic molecular features directly from compound structures and RankNet to learn how the RPLC generally orders pairs of molecules. In the retraining phase, datasets with similar chemical and chromatographic spaces to the prediction set are used as retraining reference sets to fine-tune ROASMI. This process allows finer retention information, previously scattered in the metabolomics community, to flow into the prediction sets, creating a virtuous cycle between expanding data and improving performance

Figure 2 shows the overall architecture of ROASMI: adjacent molecules are paired based on a self-defined retention interval, and then input into a Directed Message Passing Neural Network (D-MPNN) to learn features related to the retention property [33, 34]. The learned features are subsequently passed to a ranking neural network known as RankNet to learn the pH-dependent preference order [35]. The details are as follows:

##### Generating training batches as inputs

Each small molecule is encoded into isomeric SMILES via retrieval from PubChem (1 September 2024). The training pair (Ci, Cj) is defined as a small molecule Ci that eluted before Cj over a retention interval of 30 s, where the label of $\overline{{\text{P} }_{\text{ij}}}$ =1 represented that the actual probability of Ci eluting before Cj was 100%. Several training batches are generated for all training pairs with a default batch size of 64. Notably, a narrow retention interval makes the training pairs highly susceptible to noise, whereas an excessively wide interval results in a reduction in informative pairs. Here, the retention interval is recommended to be set as twice the average deviation of RTs or the average chromatographic peak width. The median RT change in the initial reference set was 18 s, and the average peak width change was 20–30 s [28].

##### Molecular representation module

This module was built upon the open-source Chemprop software, accessed at https://github.com/chemprop/chemprop. The D-MPNN was employed to translate the graph representation of molecules into continuous vectors by iteratively aggregating the features of atoms and bonds. Specifically, the SMILES encoding for each compound in (Ci, Cj)^n^ was initially converted into molecular graphs, with atoms and bonds represented as nodes and edges, respectively. Next, the graphs were encoded into computable feature vectors, where node features include the atomic type (C/N/O/P/S), number of bonds for each atom, formal charge, chirality, number of bonded hydrogens, hybridization, aromaticity, and atomic mass, whereas edge features include the bond type (single/double/triple/aromatic), conjugation, ring membership, and stereochemistry. Directed bond-based message passing operations were then applied to update higher-level hidden vectors that contained information about larger chemical substructures. The hidden vectors were ultimately summed into a single embedding representing the global molecular features.

##### Retention prediction module

The resulting embedding was fed into RankNet to learn a scoring function, where the retention scores R_i_ and R_j_ corresponded to molecules C_i_ and C_j_, respectively. Binary cross-entropy was used to compare the sigmoid of the predicted retention scores, which was the predicted probability ${{\varvec{P}}}_{{\varvec{i}}{\varvec{j}}}$ that C_i_ eluted before C_j_:

$${{\varvec{P}}}_{{\varvec{i}}{\varvec{j}}}=\frac{{{\varvec{e}}}^{{{\varvec{R}}}_{{\varvec{i}}{\varvec{j}}}}}{1+{{\varvec{e}}}^{{{\varvec{R}}}_{{\varvec{i}}{\varvec{j}}}}} {{\varvec{R}}}_{{\varvec{i}}{\varvec{j}}}={{\varvec{R}}}_{ {\varvec{J}}} -{{\varvec{R}}}_{{\varvec{I}}}$$

The consistency of P_ij_ has been demonstrated [35], as evidenced by the fact that when three analytes elute in the order C_i_, C_j_, and C_k_, if P_ij_ = 0.6 and P_jk_ = 0.6, P_ik_ was guaranteed to be greater than 0.6.

RankNet was trained by minimizing the residual between the observed and predicted probabilities, where the observed probability corresponded to the true label $\overline{{{\varvec{P}} }_{{\varvec{i}}{\varvec{j}}}}$. The loss function is expressed as:

$${{\varvec{L}}}_{{\varvec{i}}{\varvec{j}}}={-\overline{{{\varvec{P}} }_{{\varvec{i}}{\varvec{j}}}}{\varvec{l}}{\varvec{o}}{\varvec{g}}{\varvec{P}}}_{{\varvec{i}}{\varvec{j}}} -{\left(1-\overline{{{\varvec{P}} }_{{\varvec{i}}{\varvec{j}}}}\right)\mathbf{l}\mathbf{o}\mathbf{g}(1-{\varvec{P}}}_{{\varvec{i}}{\varvec{j}}})$$

#### Model development and computational infrastructure

The model implementation is based on PyTorch [36] and comprises approximately 4 million parameters, utilizing the Adam optimizer [37] with a fixed learning rate of 1e-4 and a batch size of 256. The initial reference set is randomly divided into training, validation, and testing sets in an 8:1:1 ratio, resulting in 27756, 9264, and 9228 molecule pairs, respectively. Training is performed on an NVIDIA RTX 4090 GPU (24 GB), taking approximately 7 h to complete up to 240 epochs. Early stopping is implemented to prevent overfitting, and the model is saved only when the validation accuracy for the current epoch exceeds the best validation accuracy achieved during previous epochs.

##### Hyperparameter optimization

A two-step Bayesian hyperparameter optimization was performed to improve the hyperparameters of the model: (i) the constant rank layers were maintained to find the optimal number of layers; and (ii) the optimal size of each layer was selected. The optimization results are shown in Table 1.

**Table 1:** Optimization parameters of the ROASMI

Hyperparameter	Range	Value
Number of message-passing steps	[3, 8]	6
D-MPNN hidden layer size	[300,2400]	1200
Number of rank layers	[1, 5]	3
Size of RankNet hidden layer 2	[500,1200]	800
Size of RankNet hidden layer 3	[50,500]	300
Dropout probability	[0,0.4]	0.3

##### Ensembling

The ensemble approach allowed the quantification of model uncertainty via the variance of the retention order predictions across the trained models. After a single model optimization using the initial reference set, we applied an ensemble of five models (ROASMI_1–ROASMI_5) with a fixed set of hyperparameters but different initial seeds. This follows similar uncertainty approaches in supervised learning and intrinsic clearance predictions [38, 39]. The output of the ensemble is given by the mean of the predictions, while the variance corresponds to the prediction uncertainty [40].

##### Fine-tuning

As an extension of the initial training model, retraining supports the use of multiple independent datasets as retraining reference sets. To facilitate this, modifications are made only to the “Generating training batches as inputs” section, including (i) generating training pairs for each independent dataset to avoid information confusion; (ii) creating training batches with 64 pairs per batch; and (iii) employing n-fold cross-validation to reduce overfitting caused by the small size of the retraining reference set. The training-validation data split is set as 8:2 by default.

The duration of fine-tuning varies based on factors such as the size of the retraining reference set, the training batch size, and whether a GPU is utilized. In our experiments, fine-tuning the model for A1-A12 experiments on a GPU takes approximately 10–20 min. Additionally, the model can be run on a CPU-only machine, albeit with slower computation speed. Specifically, the CPU configuration used in our experiments is an 18 vCPU AMD EPYC 9754 128-Core Processor with 60 GB of memory. On this hardware, the model's inference speed is approximately 8–10 times slower compared to GPU-based computation.

#### Comparison of molecular representations

We evaluated the generalizability of five molecular representations: (i) Rdkit_norm, which comprises 200 molecular descriptors computed by RDKit and reflects common molecular properties; (ii) Morgan_count, which includes 2048 Morgan (ECFP) counting fingerprints computed by RDKit and indicates the occurrence frequency of functional groups within a molecule; (iii) D-MPNN, which consists of a continuous vector of variable size automatically learned from molecular inputs; (iv) Hybrid_1, which is a concatenation of Morgan_count and D-MPNN; and (v) Hybrid_2, which is a concatenation of Rdkit_norm and D-MPNN [34]. The performance of these representations was compared via a held-out test set across five cross-validation folds. The generalizability of the two best-performing representations was further evaluated on independent datasets.

#### Evaluation metrics of the ROASMI

Here, we selected three metrics to evaluate the predictive performance of ROASMI: (i) pairwise prediction accuracy, which indicates the fraction of correctly classified molecule pairs within a specific dataset relative to all generated pairs [19]; (ii) Spearman correlation coefficient, a common statistic that assesses the degree of similarity between the measured and predicted retention sequences; and (iii) rank score, calculated as the arithmetic mean of the aforementioned two metrics, reflecting a combination of accuracy and correlation.

#### Visualization of ChemSS

In accordance with the Jaccard‒Tanimoto similarity computed on Morgan fingerprints, the t-distributed stochastic neighbor embedding (t-SNE) method was utilized to visualize the ChemSS between different studies [41]. A significant chemical spatial overlap between the two datasets indicated their high similarity, whereas a low overlap would suggest their differences.

#### CASMI re-annotation

**Fig. 3:**
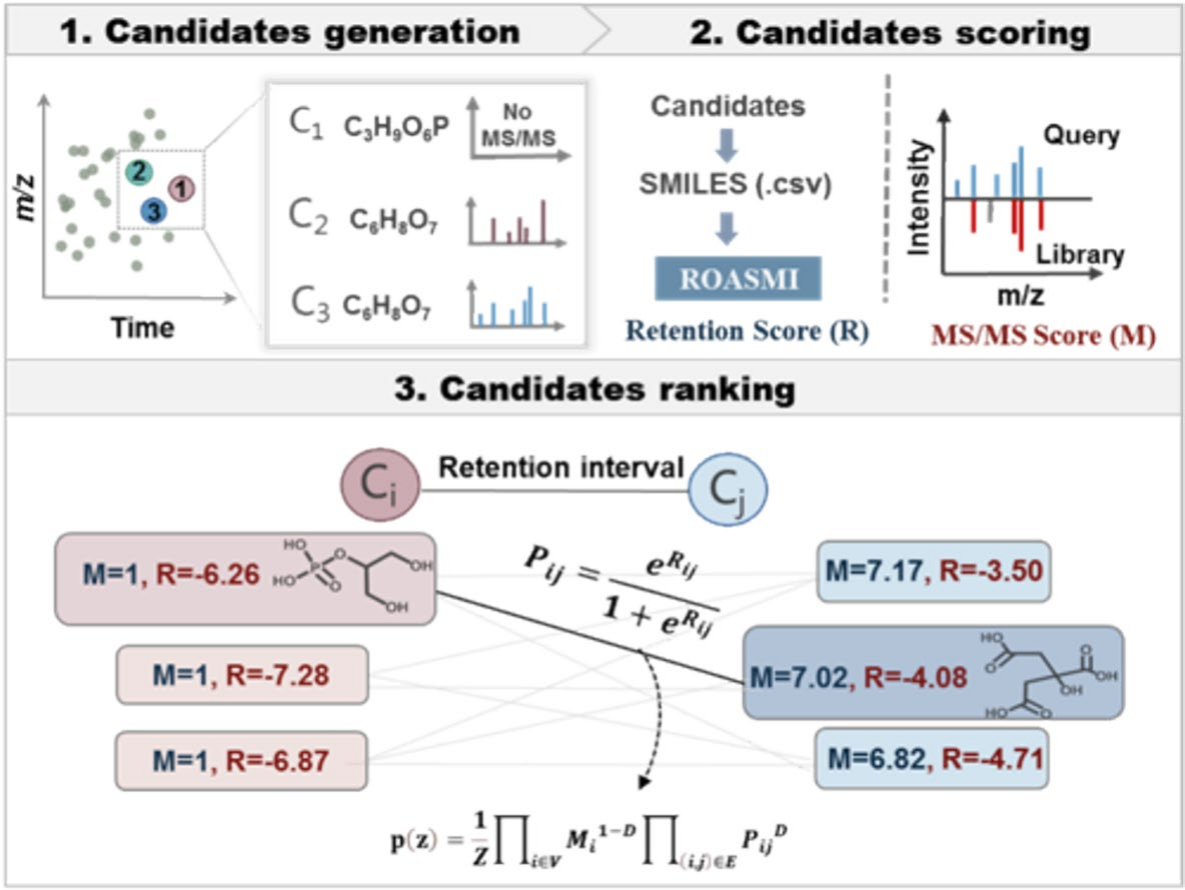
Illustration on the implement of ROASMI for small molecule identification, including the candidate generation based on inferred molecular formulae, retention and (or) MS/MS score calculation by ROASMI and (or) MS-DIAL, and candidates re-rank by integrated scores

To evaluate the applicability of ROASMI, we compared five distinct annotation strategies using the CASMI application set, including: (1) MS-DIAL alone [42], (2) SIRIUS alone [43], (3) ROASMI alone, (4) MS-DIAL combined with ROASMI, and (5) SIRIUS combined with ROASMI. Figure 3 illustrates the overall identification steps. The detailed identification steps are as follows:

##### Candidates generation

For each challenge, independent sets of candidate molecules were generated using MS-DIAL (version 4.8) and SIRIUS (version 5.8.3), respectively. MS-DIAL was configured with parameters including centroid data for MS1 and MS/MS, negative ion mode, and restrictions on formula elements to C, H, N, O, S, and P. Adducts selected included [M-H]⁻, [M-H₂O–H]⁻, [M + Na-2H]⁻, [M + Cl]⁻, [M + K-2H]⁻, and [M + FA-H]⁻. The output from MS-DIAL included candidate structure lists with names, total scores, formulas, InChIKeys, and SMILES. SIRIUS was configured with orbitrap as the instrument, maintaining the same restrictions on formula elements and adducts. The output from SIRIUS included candidate structure lists with names, CSI:FingerIDScore, formulas, InChIKeys, and SMILES.

##### Candidates scoring

For each candidate, retention score (R) was predicted using ROASMI, with the SMILES representation of candidate as input. Meanwhile, MS/MS score (M) was assigned either as a “total score” for MS-DIAL or a “CSI:FingerIDScore” for SIRIUS.

##### Candidates ranking

In annotation strategies involving MS-DIAL or SIRIUS alone, candidates were ranked solely based on MS/MS scores. When combining tools in annotation strategies (either MS-DIAL and ROASMI together or SIRIUS and ROASMI together), both MS/MS scores and retention scores were integrated to rank candidates. Specifically, we computed the maximum marginal ranking scores via the probabilistic framework developed by Eric Bach et al. [21].

The framework models score integration as an inference problem on a graphical model, where the edge $E$ corresponds to retention order prediction and the label corresponds to the predicted probability ${P}_{ij}$, the node $V$ corresponds to the candidate structure of the detected peak, and the node label corresponds to the MS/MS score ${M}_{i}$. This graph is fully connected, and random spanning tree ensembles are used to infer the maximum probability of ranking the top-k candidates for each peak, where the full vector z corresponds to the molecular candidates:

$$\text{p}\left(\text{z}\right)=\frac{1}{Z}{\prod }_{i\in V}{{M}_{i}}^{1-D}{\prod }_{\left(i,j\right)\in E}{{P}_{ij}}^{D}$$

The parameter D ∈ [0,1] controls the contribution of retention order predictions, with values set to 1 for ROASMI alone and 0.2/0.1 when combined with SIRIUS or MS-DIAL, respectively.

The position of the correct structure was noted, and top-k accuracy was used to evaluate identification performance. Correct annotations were identified when candidate structures shared the same first block of InChIKey as the ground-truth molecular structures.

## Results

### Meta-analysis reveals that pH is the critical driver of retention replicability

First, we collected independent metabolite datasets that met the following criteria: binary-solvent mobile phase, one-dimensional RP column, and at least 30 MSI level 1 identifications. We then paired datasets containing more than 30 duplicate compounds and calculated the corresponding CCRS values to evaluate the replicability of the retention sequences. Finally, we investigated the influence of the eluent pH, organic solvent type, column temperature, and column chemistry on the CCRS.

**Fig. 4:**
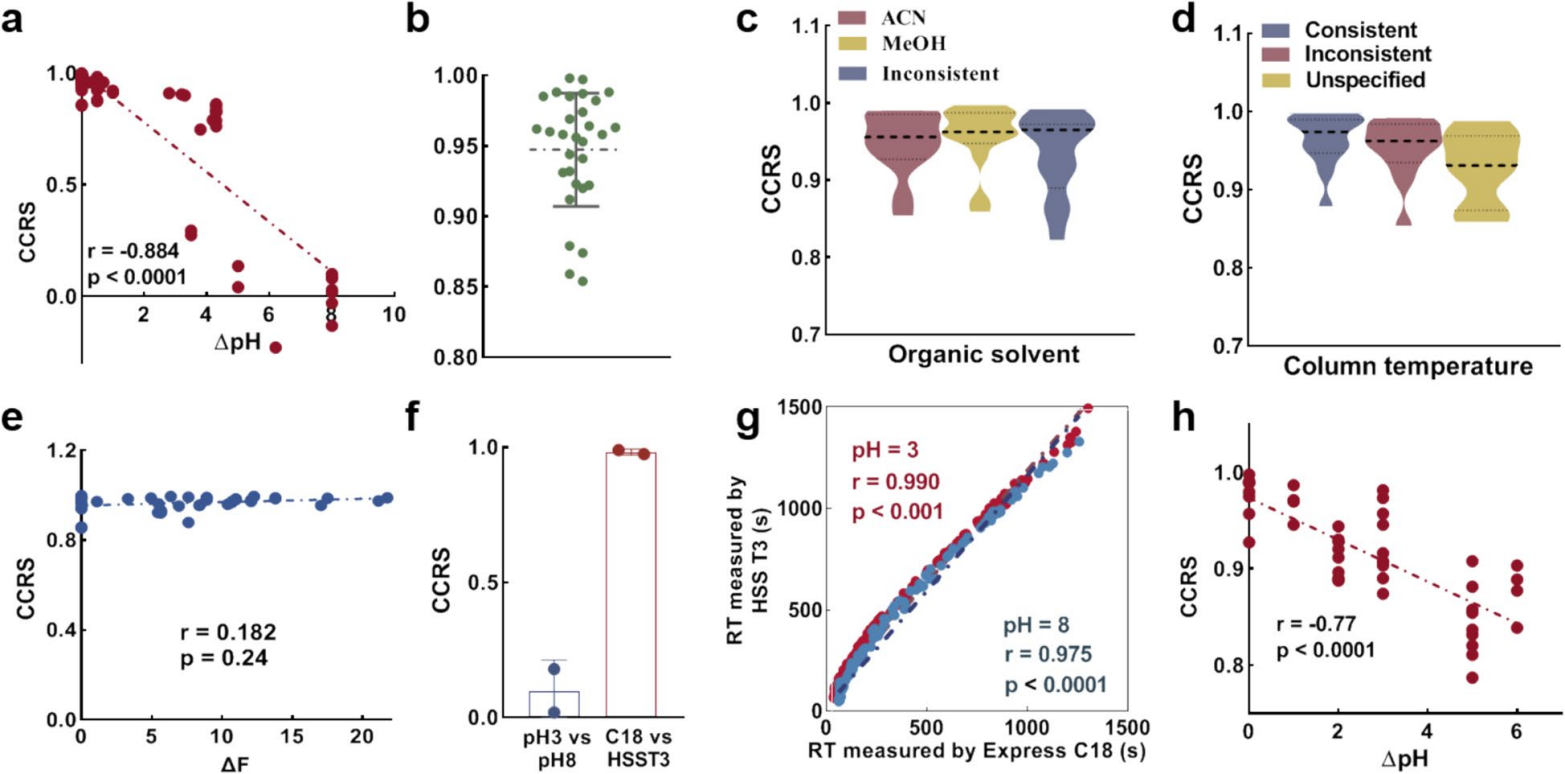
pH is a critical factor driving the replicability of retention sequences. (**a**) Scatter plot of ΔpH versus CCRS for 59 pairs of datasets. r value denotes the Spearman correlation between ΔpH and CCRS (95% CI − 0.931 to − 0.809). (**b**) Statistics of CCRS values for 33 pairs of datasets with ΔpH ≤ 1, presented as the mean ± s.d. (**c**) Violin plots of the CCRS values under varying organic phases (n ≥ 13) and (**d**) column temperatures (n ≥ 10). The lower and upper hinges represent the first and third quartiles, respectively, whereas the centerline indicates the median and the mean values. (**e**) Spearman correlation between ΔF and CCRS for 43 pairs of datasets, with the 95% confidence interval for r ranging from − 0.134 to 0.465. (**f**) Comparison of the effects of pH and column on CCRS using four representative pairs of datasets. The data are presented as the means ± s.d. (**g**) Spearman correlation between RT measured by the Express C18 and HSS T3 columns under two pH conditions: the blue dots and dashed line indicate the scenario at pH 8 (*dataset_65* vs. *dataset_77*, n = 207), with r = 0.975 (95% CI 0.967 to 0.981); the red dots and dashed line indicate the scenario at pH 3 (*dataset_ 66* vs. *dataset_71*, n = 197), with r = 0.990 (95% CI 0.987 to 0.993). All p values were determined via a two-tailed unpaired test. (**h**) Scatter plot of the ΔpH versus CCRS for one study involving multiple chromatographic conditions. The r value denotes the Spearman correlation between ΔpH and CCRS (95% CI − 0.869 to − 0.613)

It has been reported that the RT of a compound significantly changes when the pH of the buffer solution is within ± 1.5 units of the compound’s pKa, indicating the critical role of pH in retention behavior [44–46]. Similarly, we observed a significant negative correlation between the CCRS and the ΔpH in 59 paired datasets (Fig. 4a). When the pH difference (∆pH) reached five, the retention sequences became nearly entirely nonreplicable. Conversely, among the 30 pairs of studies with ∆pH ≤ 1, the average CCRS value was remarkably high at 0.95, with only four pairs falling below 0.9 (Fig. 4b).

Previous research has shown that differences in the organic eluents, gradient, and specific C18 columns do not significantly affect the largely replicable retention order [15]. Our results further confirmed this observation. When ∆pH was maintained within 1, the retention sequences between paired datasets remained highly replicable, regardless of differences in organic solvent (Fig. 4c) or column temperature (Fig. 4d). Upon examining 43 pairs of datasets with ∆pH values less than 1, we found no correlation between CCRS and F difference (∆F), which serves as an indicator of similarity between two columns (Fig. 4e). We also conducted additional analyses that included three independent comparisons: the first involved four studies conducted within the same laboratory, where only the pH or column type varied between pairs (Fig. 4f, g); the second encompassed multiple chromatographic conditions, incorporating various eluent compositions with pH values ranging from 2.1 to 10.0 [47] (Fig. 4h); and the third consisted of five interlaboratory datasets featuring 12 overlapping compounds (Supplementary Fig. 1).

Together, these results suggest that eluent pH is the main factor affecting the replicability of retention order in the RPLC system. Importantly, the above findings do not negate the role of other LC conditions in changing the chromatographic selectivity. For example, tools such as multifactorial peak crossover (MPC) can switch the retention order of target analytes by adjusting the column temperature, mobile composition, and gradient slope [48]. However, these tools are mainly used for CM development and optimization, provided that standards or physicochemical parameters for the target analytes are available. When developing retention models designed to assist in small molecule identification, chromatographic theory is often simplified to accommodate the large number of peaks that need to be identified [49].

### Learning replicable retention order via ROASMI

Since analytes with different CMs but maintaining similar pH values can be expected to have a replicable retention order, we hypothesized that ROASMI could learn generic retention information from *dataset_87*, which contains 50,447 RT records and a widely used pH value of 2.68. Unlike regression, we utilized a learning-to-rank module, RankNet, to estimate the probability of the elution order of paired compounds. The prediction probabilities from multiple models within an ensemble were then averaged to improve robustness. Its neural network architecture embraces a two-phase training strategy, thus allowing user to fine-tune the initial ROASMI model via retraining reference sets to achieve satisfactory prediction.

#### Comparison of the molecular embedding modules

**Fig. 5:**
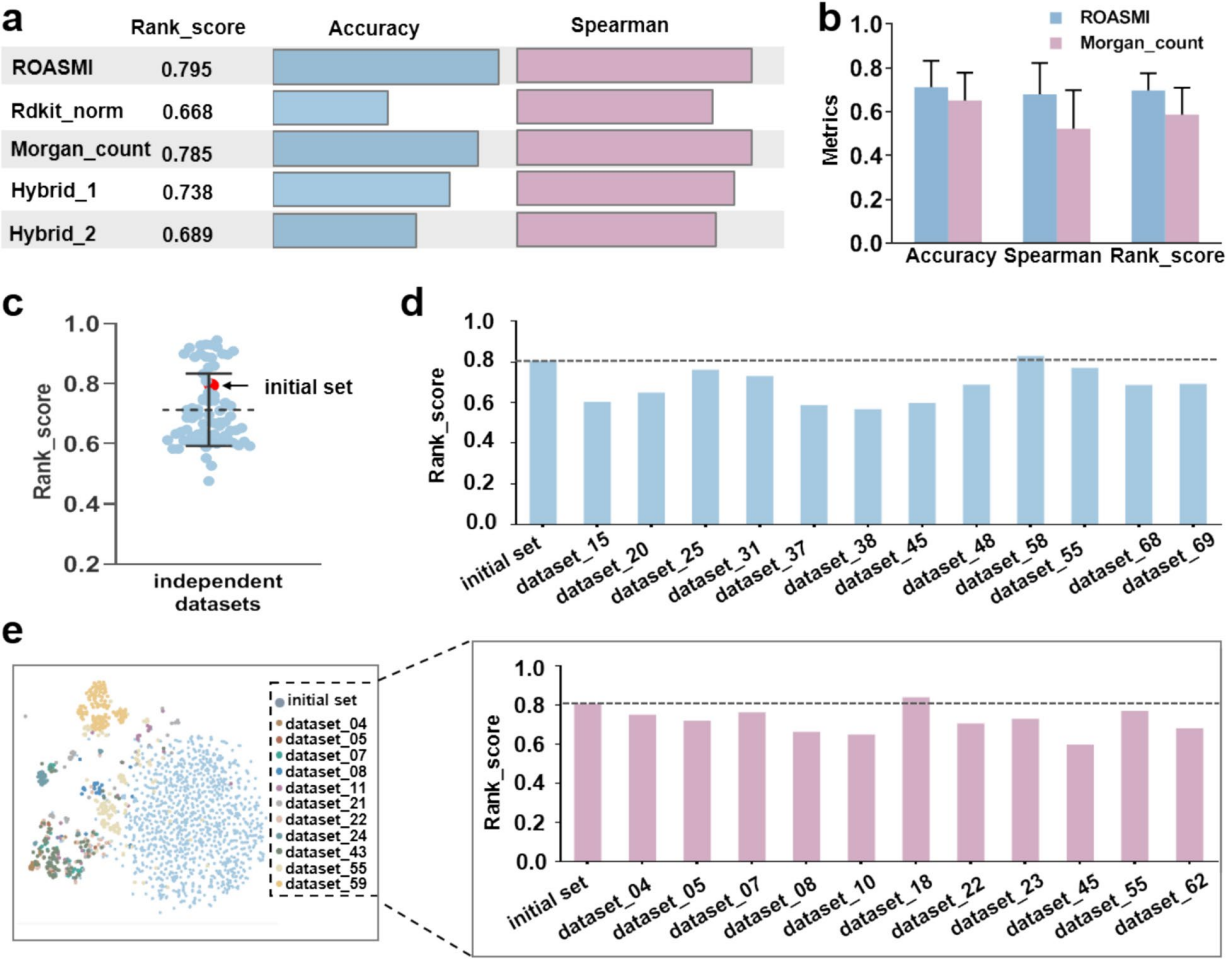
Performance evaluation of ROASMI on independent datasets. (**a**) Comparisons of the molecular representation modules in the initial training stage. (**b**) Averaged generalizability of ROASMI and Morgan_count in 71 independent datasets. (**c**) Predictive performance of ROASMI on the initial reference set and 71 independent datasets. The data are presented as the means ± s.d. (**d** and **e**) The generalizability of ROASMI across diverse chromatographic (**d**) and chemical spaces (**e**). The dashed line indicates the performance benchmark of the initial reference set

Figure 5a shows the average performance in the initial training stage for different molecular representation approaches. Compared with the performance of ROASMI between two hybrid characterizations (Hybrid_1,2), we observed that ROASMI alone could extract sufficient, nonredundant retention information. Although ROASMI and Morgan_count had similar training performances, ROASMI performed better in most cases on independent datasets across 71 independent datasets with similar pH values to the initial reference set (Fig. 5b, Table 2).

**Table 2:** Comparison of the generalizability of the two models on independent datasets

Dataset_ID	ROASMI			Morgan_count		
	Accuracy	Spearman	Rank score	Accuracy	Spearman	Rank score
initial_set	0.72	0.87	0.795	0.7	0.871	0.786
*dataset_01*	0.923	0.698	0.811	0.846	0.607	0.727
*dataset_02*	0.889	0.91	0.900	0.667	0.647	0.657
*dataset_03*	0.809	0.418	0.614	0.524	0.3	0.412
*dataset_04*	0.647	0.852	0.750	0.559	0.533	0.546
*dataset_05*	0.741	0.698	0.720	0.556	0.25	0.403
*dataset_06*	0.719	0.5	0.610	0.656	0.577	0.617
*dataset_07*	0.625	0.898	0.762	0.625	0.65	0.638
*dataset_08*	0.714	0.612	0.663	0.574	0.204	0.389
*dataset_10*	0.583	0.715	0.649	0.417	0.532	0.475
*dataset_11*	0.737	0.905	0.821	0.763	0.849	0.806
*dataset_12*	0.476	0.71	0.593	0.524	0.434	0.479
*dataset_17*	0.64	0.85	0.745	0.6	0.28	0.440
*dataset_18*	0.833	0.844	0.839	0.694	0.521	0.608
*dataset_19*	0.625	0.81	0.718	0.667	0.57	0.619
*dataset_20*	0.609	0.688	0.649	0.5	0.299	0.400
*dataset_21*	0.692	0.677	0.685	0.564	0.342	0.453
*dataset_22*	0.608	0.802	0.705	0.45	0.38	0.415
*dataset_23*	0.66	0.798	0.729	0.54	0.497	0.519
*dataset_25*	0.854	0.668	0.761	0.854	0.611	0.733
*dataset_26*	0.745	0.734	0.740	0.596	0.374	0.485
*dataset_27*	0.6	0.561	0.581	0.48	0.38	0.430
*dataset_28*	0.639	0.811	0.725	0.623	0.809	0.716
*dataset_29*	0.674	0.3	0.487	0.63	0.164	0.397
*dataset_30*	0.632	0.777	0.705	0.611	0.676	0.644
*dataset_31*	0.75	0.71	0.730	0.813	0.569	0.691
*dataset_32*	0.603	0.547	0.575	0.466	0.444	0.455
*dataset_33*	0.61	0.586	0.598	0.593	0.437	0.515
*dataset_34*	0.615	0.7	0.658	0.538	0.496	0.517
*dataset_35*	0.667	0.595	0.631	0.617	0.566	0.592
*dataset_36*	0.645	0.729	0.687	0.645	0.536	0.591
*dataset_37*	0.727	0.446	0.587	0.545	0.391	0.468
*dataset_38*	0.744	0.39	0.567	0.698	0.397	0.548
*dataset_39*	0.607	0.575	0.591	0.589	0.574	0.582
*dataset_40*	0.672	0.663	0.668	0.625	0.524	0.575
*dataset_41*	0.583	0.844	0.714	0.708	0.818	0.763
*dataset_42*	0.527	0.696	0.612	0.5	0.34	0.420
*dataset_43*	0.909	0.689	0.799	1	0.729	0.865
*dataset_44*	0.9	0.68	0.79	0.84	0.39	0.615
*dataset_45*	0.697	0.498	0.598	0.636	0.312	0.474
*dataset_46*	0.612	0.824	0.718	0.612	0.605	0.609
*dataset_47*	0.687	0.7	0.694	0.6	0.493	0.547
*dataset_48*	0.9	0.474	0.687	0.85	0.409	0.630
*dataset_51*	0.595	0.846	0.721	0.75	0.797	0.774
*dataset_52*	0.697	0.764	0.731	0.64	0.697	0.669
*dataset_53*	0.71	0.599	0.655	0.548	0.252	0.400
*dataset_54*	0.631	0.681	0.656	0.544	0.627	0.586
*dataset_55*	0.686	0.854	0.770	0.62	0.646	0.633
*dataset_56*	0.762	0.712	0.737	0.743	0.58	0.662
*dataset_58*	0.945	0.712	0.829	0.956	0.636	0.796
*dataset_59*	0.633	0.767	0.700	0.695	0.72	0.708
*dataset_60*	0.603	0.356	0.480	0.543	0.238	0.391
*dataset_62*	0.713	0.648	0.681	0.627	0.473	0.550
*dataset_63*	0.771	0.497	0.634	0.771	0.476	0.624
*dataset_64*	0.611	0.77	0.691	0.585	0.762	0.674
*dataset_66*	0.931	0.648	0.790	0.931	0.601	0.766
*dataset_67*	0.605	0.822	0.714	0.558	0.746	0.652
*dataset_68*	0.589	0.782	0.686	0.589	0.693	0.641
*dataset_69*	0.552	0.83	0.691	0.596	0.746	0.671
*dataset_71*	0.897	0.689	0.793	0.911	0.652	0.782
*dataset_72*	0.86	0.43	0.645	0.63	0.35	0.490
*dataset_73*	0.593	0.792	0.693	0.58	0.753	0.667
*dataset_74*	0.89	0.45	0.67	0.68	0.37	0.525
*dataset_75*	0.928	0.575	0.752	0.867	0.415	0.641
*dataset_76*	0.92	0.52	0.72	0.57	0.24	0.405
*dataset_78*	0.93	0.55	0.74	0.7	0.19	0.445
*dataset_79*	0.9	0.656	0.778	0.859	0.595	0.727
*dataset_80*	0.887	0.649	0.768	0.87	0.585	0.728
*dataset_83*	0.627	0.767	0.697	0.614	0.727	0.671
*dataset_84*	0.669	0.722	0.696	0.625	0.559	0.592
*dataset_85*	0.653	0.841	0.747	0.606	0.706	0.656
*dataset_86*	0.644	0.825	0.735	0.617	0.79	0.704

(should appear in above the “Generalizability assessments of ROASMI” section on page 17 in the text during production)

#### Generalizability assessments of ROASMI

As shown in Table 2 , ROASMI has excellent generalizability across 71 RPLC datasets, with ΔpH values within ± 1 of the initial reference set. Remarkably, ROASMI displayed an average rank score of 0.696 on these independent datasets, whereas the initial reference set's rank score was 0.795 (Fig. 5c). Even for datasets with significantly different chromatographic spaces, ROASMI maintained high rank scores aligned with those of the initial reference set (Fig. 5d). This consistent performance also extended to chemical spaces, indicating ROASMI's ability to accurately predict the retention property for small molecules not present in the initial reference sets (Fig. 5e).

For these 71 datasets, we performed an exploratory analysis of the relationship between ROASMI performance and individual chromatographic conditions. As shown in Supplementary Fig. 2, these datasets cover a wide range of chromatographic conditions. However, in this study at least, we did not find any significant functional relationships between ROASMI performance and chromatographic factors other than pH.

We also examined the relationship between model performance and compound properties. We used the difference between the predicted ranking and the true ranking to measure the predictive performance of the model for each compound. Focusing on the top-performing (top 10%) and bottom-performing (bottom 10%) compound sets, we obtained their physical properties from PubChem. The results, presented in Supplementary Fig. 3a, b, show significant differences in Hydrogen Bond Donor Count (HBDC) and Hydrogen Bond Acceptor Count (HBAC) distributions between the top 10% and bottom 10% compound sets (P < 0.0001 for both HBDC and HBAC, Mann–Whitney test). However, it is essential to note that this observation does not imply a causal relationship between higher values of HBDC and HBAC and poorer model performance. We also found significant differences in XlogP values and Heavy Atom Count between the top 10% and bottom 10% compounds (Supplementary Fig. 3c, d; P < 0.001 for XlogP and P < 0.0001 for Heavy Atom Count, Mann–Whitney test). These findings imply to some extent that the properties of the compounds themselves have a more intricate impact on model performance than the chromatographic conditions.

#### Retraining performance of ROASMI

The superior generalizability of the initial ROASMI placed high demands on the choice of retraining reference sets. To be realistic, we assume that a single dataset is used as the retraining reference set and evaluate the transferability of ROASMI in terms of two metrics, ChemSS and ChrSS. As shown in Table 3, we observed substantial improvements in the rank scores only when novel reference sets shared overlapping chemical and chromatographic spaces with prediction sets. For example, the rank scores of A1 and A2 increased by 0.21 and 0.1, respectively, whereas the improvements in A11 and A12 were negligible (Fig. 5d, e). In addition, the amount of data in the reference set has less impact on the retraining performance. On the one hand, ROASMI can be effectively fine-tuned even with a relatively small amount of data, such as the 361 reference compounds used in A1 and the 110 reference compounds used in A4. In A10, on the other hand, *dataset_86* has a total of 1955 compounds, and we randomly selected 10–90% of the small molecule fractions as the reference set and found that the increase in data volume did not further improve the rank score (Supplementary Fig. 4).

**Table 3:** Retraining results for the RPLC datasets

Experiment no	Retraining Reference set	Prediction set	ChemSS	ChrSS	Initial train	Retrain	△score^*^
A1	*dataset_74*	*dataset_72*	0.89	4	0.65	0.86	0.21
A2	*dataset_80*	*dataset_79*	0.99	4	0.78	0.88	0.10
A3	*dataset_71*	*dataset_66*	0.94	4	0.79	0.89	0.10
A4	*RP12 pos*	*RP22 pos*	0.94	3	0.79	0.89	0.10
A5	*dataset_68*	*dataset_51*	0.01	2	0.72	0.81	0.09
A6	*dataset_76*	*dataset_78*	0.78	4	0.74	0.80	0.06
A7	*dataset_84*	*dataset_68*	0	4	0.69	0.72	0.03
A8	*dataset_73*	*dataset_19*	0.15	2	0.72	0.74	0.03
A9	*dataset_68*	*dataset_42*	0.09	3	0.61	0.64	0.03
A10	*dataset_86_90%train*	*dataset_86_10%test*	0	5	0.73	0.77	0.03
A11	*dataset_84*	*dataset_42*	0	3	0.61	0.63	0.02
A12	*CASMI_2016_train*	*CASMI_2016_challenge*	0	5	0.65	0.65	0.00

^*^∆ score represents the difference in rank scores between the initial and retrained models

#### Applicability assessments of ROASMI

We evaluated the applicability of ROASMI in three tests on four application sets with varying complexities and samples. In real-world small-molecule identification, retention scores and MS/MS scores can be used either alone or in combination to annotate peaks. Here, we utilized initial ROASMI to predict retention scores for candidate molecules and used SIRIUS and MS-DIAL, which are the best-performing automated annotation tools in the 2022 CASMI challenge, to predict MS/MS scores.

**Fig. 6:**
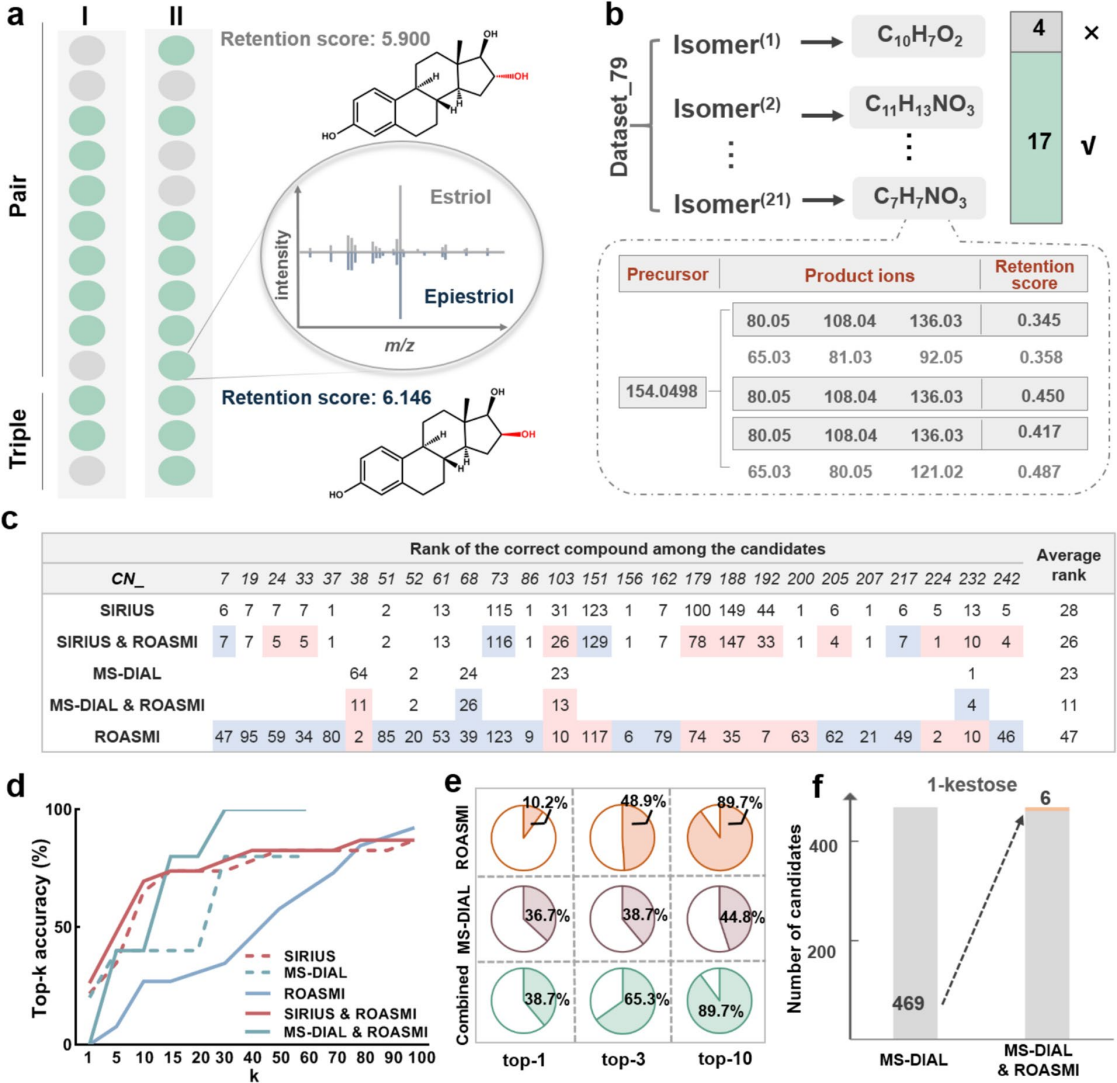
Applicability assessments of ROASMI. (**a**) Identification results of the original compound-specific model (I) and ROASMI (II). The green portions indicate correct discriminations, whereas the gray portions indicate incorrect discriminations. (**b**) ROASMI's ability to identify a high fraction of isomers. Comparison of candidate ranking (**c**) and top-k accuracy (**d**) in the 2022 CASMI competition using different models. For each CN (challenge number), using SIRIUS or MS-DIAL alone as a baseline, the correct compound ranking forward shifts are labeled in red, and backward shifts are labeled in blue. (**e**) Candidate ranking comparison using different models. (**f**) Rank improvement after filtering by the retention score, using 1-ketose as an example

In the first test, we assessed the ability to discriminate isomers via retention scores alone. We chose two metabolomic datasets differing in the amount and distribution of isomer groups. The human metabolomic *dataset_71* contained 13 isomeric pairs and triplets, whereas the gut bacteria-derived metabolomic *dataset_79* contained 19 pairs and triplets and an additional three groups of quadruplets and quintuplets. Using the predicted retention scores, ROASMI successfully distinguished the vast majority of isomers in both datasets, outperforming the customized retention model of *dataset_71* (Fig. 6a, b, Supplementary Table 1). Notably, ROASMI successfully distinguished between estriol and epiestriol in *dataset_71* (Fig. 6a), as did 3-amino-4-hydroxybenzoic acid, 3-hydroxyanthranilic acid, and 4-aminosalicylic acid in *dataset_79* (Fig. 6b, Supplementary Table 2). These isomers have nearly identical MS/MS data and are difficult to distinguish accurately via MS/MS scorers.

In the second test, we evaluated the annotation performance of combining ROAMSI with MS/MS scorers by reannotating 106 priority challenges from the 2022 CASMI. The challenge was considered a hit if a structure in the candidate list matched the first block of the InChI Key for the correct structure. Challenges processed with SIRIUS and MS-DIAL yielded 25 and five hits, respectively. Figure 6c, d present the annotation results for these hits. Despite the strong baseline performance of SIRIUS, ROASMI improved the top-1 and top-5 accuracies by 1 and 3 units, respectively. Compared with the use of SIRIUS alone and MS-DIAL alone, the combined use of ROASMI improved the average ranking of the correct structure in the candidate list by 2 and 12 units, respectively. Moreover, for compounds such as 'CN_188' and 'CN_192', which lack standard MS/MS spectra in MS libraries, using retention scores yielded better top-k accuracies than did using experimental MS/MS spectra.

In the third test, we used a plant-derived metabolic dataset (*dataset_38*) to replicate a real-world situation as closely as possible. Among the 49 peaks with correctly inferred formulas, 23 lacked meaningful MS/MS spectra. This situation is common in samples with low-abundance peaks and large-scale metabolomics studies [5, 6]. Supplementary Table 3 records the annotation results of ROASMI alone, MS-DIAL alone, and the combined use of both tools. Similar to the second test, the combined approach achieved the highest top-1 and top-3 identification accuracies, confirming the complementary nature of the retention and spectral information (Fig. 6e). Furthermore, for the 23 peaks that MS-DIAL failed to annotate, ROASMI reduced the average number of candidate compounds by 87.3%. For example, in the case of 1-ketose, ROASMI elevated the ranking of the correct compound to the sixth position out of 469 unweighted potential candidates (Fig. 6f).

## Discussion

In untargeted metabolomics studies, structural annotation typically leads to a level 4 or 5 annotation for detected peaks lacking MS/MS spectra [50]. However, ROASMI's predictive retention scores offer an alternative approach to infer the structure of these peaks, reducing the reliance on experimental MS/MS data. The practical utility of ROASMI is exemplified through real-world metabolomics cases of varying complexities, where it seamlessly integrates with existing MS/MS tools, resulting in highly accurate annotations. Additionally, repurposing heterogeneous retention data from the metabolomics community, characterized on the basis of peak‒peak relationships under similar pH conditions, has proven valuable in supporting compound identification.

The primary challenge in developing retention-based annotation tools is ensuring their generalizability to independent datasets. A single external validation set or data split from the training set is inadequate to reflect the model’s generalizability. Here, 71 independent datasets with diverse chromatographic and chemical spaces were used as external validation sets. The generalizability of ROASMI is evidenced by its performance on these datasets, which is comparable to that on the initial reference set, all without the need for laborious calibrations or projections.

Acknowledging the imperfections of machine learning approaches and emphasizing the importance of integration between these approaches and solid mechanistic understanding is crucial. Our comprehensive framework for modeling retention behavior considers three main factors: uncontrollable, kinetic, and thermodynamic factors [14]. Strategies such as strict dataset inclusion and the introduction of “retention intervals” effectively mitigate the impact of uncontrollable factors. Addressing kinetic factors involves changing the prediction target from pointwise RT to pairwise elution order. For thermodynamic factors, the use of well-annotated datasets with similar pH conditions as reference sets facilitates the propagation of retention information to datasets requiring annotation.

When using ROASMI in practical applications, it is important to consider molecular pairs that are indistinguishable by the current model. To illustrate this, we take two pairs of compounds from dataset_2 as examples. The first pair consists of 4-hydroxybenzoic acid (peak 1, RT = 5.41 min) and rutin (peak 2, RT = 6.44 min). ROASMI predicts that peak 1 elutes before peak 2 with an 81% probability and a variance of 9%. This indicates that the model is confident in its prediction, suggesting that the two peaks are distinguishable. The second pair involves 4-hydroxybenzoic acid (peak 1, RT = 5.41 min) and 2,5-dihydroxybenzoic acid (peak 3, RT = 5.48 min). According to the model prediction, the probability of peak 1 eluting before peak 3 is 54% with a variance of 25%. The probability being close to 50% and the large variance imply that the model is unable to distinguish the relative order of the two peaks (i.e., indistinguishable), which is close to the actual situation. In reality, when the chromatographic peaks of two compounds are close enough together, their order of retention may not be consistent across experiments. Therefore, this rough assessment based on the ensemble model partially serves the purpose of identifying which molecular pairs are indistinguishable in terms of retention order. Such pairs should be excluded from the subsequent small molecule identification process.

Future retention-based annotation tools would benefit from coordinated updates and contributions from the research community, including initiatives such as data sharing [24], faithful annotation reporting, and the creation of search tools. The current version of ROASMI applies to conventional compounds within the elemental restrictions of C, H, N, O, P, and S, leaving a vast chemical space yet to be explored. Furthermore, ROASMI currently applies only to RPLC systems, which limits its application to a specific chromatographic space. Compared with RPLC, the separation mechanism of hydrophilic interaction liquid chromatography (HILIC) is much more complex, involving not only partitioning but also secondary interactions, such as electrostatic interactions. Constructing retention models with generalizability under the HILIC system is our future endeavor. Additionally, addressing RT outliers resulting from slight changes in experimental conditions requires in-depth mechanistic studies.

## Supplementary Information

Additional file 1

## Data Availability

The data supporting the conclusions of this article is available from GitHub (https://github.com/FangYuan717/ROASMI) and archived in the Zenodo repository (https://doi.org/10.5281/zenodo.13927187).

## References

[1] Alseekh S, Aharoni A, Brotman Y, Contrepois K, D’Auria J, Ewald J, J CE, Fraser PD, Giavalisco P, Hall RD et al (2021) Mass spectrometry-based metabolomics: a guide for annotation, quantification and best reporting practices. Nat Methods 18:747–756. 10.1038/s41592-021-01197-1 34239102 10.1038/s41592-021-01197-1 PMC8592384

[2] Gauglitz JM, West KA, Bittremieux W, Williams CL, Weldon KC, Panitchpakdi M, Di Ottavio F, Aceves CM, Brown E, Sikora NC et al (2022) Enhancing untargeted metabolomics using metadata-based source annotation. Nat Biotechnol 40:1774–1779. 10.1038/s41587-022-01368-1 35798960 10.1038/s41587-022-01368-1 PMC10277029

[3] Goldman S, Wohlwend J, Stražar M, Haroush G, Xavier RJ, Coley CW (2023) Annotating metabolite mass spectra with domain-inspired chemical formula transformers. Nat Mach Intell 5:965–979. 10.1038/s42256-023-00708-3

[4] Jarmusch SA, van der Hooft JJJ, Dorrestein PC, Jarmusch AK (2021) Advancements in capturing and mining mass spectrometry data are transforming natural products research. Nat Prod Rep 38:2066–2082. 10.1039/d1np00040c 34612288 10.1039/d1np00040c PMC8667781

[5] Viant MR, Kurland IJ, Jones MR, Dunn WB (2017) How close are we to complete annotation of metabolomes? Curr Opin Chem Biol 36:64–69. 10.1016/j.cbpa.2017.01.001 28113135 10.1016/j.cbpa.2017.01.001 PMC5337156

[6] Chaleckis R, Meister I, Zhang P, Wheelock CE (2019) Challenges, progress and promises of metabolite annotation for LC-MS-based metabolomics. Curr Opin Biotechnol 55:44–50. 10.1016/j.copbio.2018.07.010 30138778 10.1016/j.copbio.2018.07.010

[7] Sagandykova G, Buszewski B (2021) Perspectives and recent advances in quantitative structure-retention relationships for high performance liquid chromatography. How far are we? TrAC Trends Anal Chem. 10.1016/j.trac.2021.116294

[8] Tian Z, Liu F, Li D, Fernie AR, Chen W (2022) Strategies for structure elucidation of small molecules based on LC-MS/MS data from complex biological samples. Comput Struct Biotechnol J 20:5085–5097. 10.1016/j.csbj.2022.09.004 36187931 10.1016/j.csbj.2022.09.004 PMC9489805

[9] Novoa-del-Toro EM, Witting M (2024) Navigating common pitfalls in metabolite identification and metabolomics bioinformatics. Metabolomics. 10.1007/s11306-024-02167-2 10.1007/s11306-024-02167-2 PMC11416380 39305388

[10] Han S, Van Treuren W, Fischer CR, Merrill BD, DeFelice BC, Sanchez JM, Higginbottom SK, Guthrie L, Fall LA, Dodd D et al (2021) A metabolomics pipeline for the mechanistic interrogation of the gut microbiome. Nature 595:415–420. 10.1038/s41586-021-03707-9 34262212 10.1038/s41586-021-03707-9 PMC8939302

[11] Olivier-Jimenez D, Chollet-Krugler M, Rondeau D, Beniddir MA, Ferron S, Delhaye T, Allard PM, Wolfender JL, Sipman HJM, Lucking R et al (2019) A database of high-resolution MS/MS spectra for lichen metabolites. Sci Data 6:294. 10.1038/s41597-019-0305-1 31780665 10.1038/s41597-019-0305-1 PMC6882832

[12] Martínez-Domínguez G, Romero-González R, Garrido Frenich A (2016) Multiclass methodology to determine pesticides and mycotoxins in green tea and royal jelly supplements by liquid chromatography coupled to Orbitrap high resolution mass spectrometry. Food Chem 197:907–915. 10.1016/j.foodchem.2015.11.070 26617033 10.1016/j.foodchem.2015.11.070

[13] National Academies of Sciences, E., Medicine, Policy, Global, A., Committee on Science, E.M., Public, P., Board on Research, D., Information, Division on, E., Physical, S., et al. (2019). Reproducibility and Replicability in Science. In (National Academies Press (US) Copyright 2019 by the National Academy of Sciences. All rights reserved.). 10.17226/25303.

[14] Eugster PJ, Boccard J, Debrus B, Breant L, Wolfender JL, Martel S, Carrupt PA (2014) Retention time prediction for dereplication of natural products (CxHyOz) in LC-MS metabolite profiling. Phytochemistry 108:196–207. 10.1016/j.phytochem.2014.10.005 25457501 10.1016/j.phytochem.2014.10.005

[15] Stanstrup J, Neumann S, Vrhovsek U (2015) PredRet: prediction of retention time by direct mapping between multiple chromatographic systems. Anal Chem 87:9421–9428. 10.1021/acs.analchem.5b02287 26289378 10.1021/acs.analchem.5b02287

[16] Bouwmeester R, Martens L, Degroeve S (2020) Generalized calibration across liquid chromatography setups for generic prediction of small-molecule retention times. Anal Chem 92:6571–6578. 10.1021/acs.analchem.0c00233 32281370 10.1021/acs.analchem.0c00233

[17] Witting M, Bocker S (2020) Current status of retention time prediction in metabolite identification. J Sep Sci 43:1746–1754. 10.1002/jssc.202000060 32144942 10.1002/jssc.202000060

[18] Garcia CA, Gil-de-la-Fuente A, Barbas C, Otero A (2022) Probabilistic metabolite annotation using retention time prediction and meta-learned projections. J Cheminform 14:33. 10.1186/s13321-022-00613-8 35672784 10.1186/s13321-022-00613-8 PMC9172150

[19] Bach E, Szedmak S, Brouard C, Bocker S, Rousu J (2018) Liquid-chromatography retention order prediction for metabolite identification. Bioinformatics 34:i875–i883. 10.1093/bioinformatics/bty590 30423079 10.1093/bioinformatics/bty590

[20] Liu JJ, Alipuly A, Bączek T, Wong MW, Žuvela P (2019) Quantitative structure-retention relationships with non-linear programming for prediction of chromatographic elution order. Int J Mol Sci. 10.3390/ijms20143443 10.3390/ijms20143443 PMC6678770 31336981

[21] Bach E, Rogers S, Williamson J, Rousu J (2021) Probabilistic framework for integration of mass spectrum and retention time information in small molecule identification. Bioinformatics 37:1724–1731. 10.1093/bioinformatics/btaa998 33244585 10.1093/bioinformatics/btaa998 PMC8289373

[22] Bach E, Schymanski EL, Rousu J (2022) Joint structural annotation of small molecules using liquid chromatography retention order and tandem mass spectrometry data. Nat Mach Intell 4:1224–1237. 10.1038/s42256-022-00577-2

[23] Boelrijk J, van Herwerden D, Ensing B, Forré P, Samanipour S (2023) Predicting RP-LC retention indices of structurally unknown chemicals from mass spectrometry data. J Cheminform. 10.1186/s13321-023-00699-8 10.1186/s13321-023-00699-8 PMC9960388 36829215

[24] Kretschmer F, Harrieder E-M, Hoffmann MA, Böcker S, Witting M (2024) RepoRT: a comprehensive repository for small molecule retention times. Nat Methods 21:153–155. 10.1038/s41592-023-02143-z 38191934 10.1038/s41592-023-02143-z

[25] Sud M, Fahy E, Cotter D, Azam K, Vadivelu I, Burant C, Edison A, Fiehn O, Higashi R, Nair KS et al (2016) Metabolomics workbench: an international repository for metabolomics data and metadata, metabolite standards, protocols, tutorials and training, and analysis tools. Nucleic Acids Res 44:D463-470. 10.1093/nar/gkv1042 26467476 10.1093/nar/gkv1042 PMC4702780

[26] Haug K, Cochrane K, Nainala VC, Williams M, Chang J, Jayaseelan KV, O’Donovan C (2020) MetaboLights: a resource evolving in response to the needs of its scientific community. Nucleic Acids Res 48:D440–D444. 10.1093/nar/gkz1019 31691833 10.1093/nar/gkz1019 PMC7145518

[27] Harrieder E-M, Kretschmer F, Dunn W, Böcker S, Witting M (2022) Critical assessment of chromatographic metadata in publicly available metabolomics data repositories. Metabolomics. 10.1007/s11306-022-01956-x 10.1007/s11306-022-01956-x PMC9701651 36436113

[28] Domingo-Almenara X, Guijas C, Billings E, Montenegro-Burke JR, Uritboonthai W, Aisporna AE, Chen E, Benton HP, Siuzdak G (2019) The METLIN small molecule dataset for machine learning-based retention time prediction. Nat Commun 10:5811. 10.1038/s41467-019-13680-7 31862874 10.1038/s41467-019-13680-7 PMC6925099

[29] Bruderer T, Varesio E, Hopfgartner G (2017) The use of LC predicted retention times to extend metabolites identification with SWATH data acquisition. J Chromatogr B Analyt Technol Biomed Life Sci 1071:3–10. 10.1016/j.jchromb.2017.07.016 10.1016/j.jchromb.2017.07.016 28780068

[30] CASMI2022: Critical Assessment of Small Molecule Identification. (2022). http://www.casmi-contest.org/2022/index.shtml.

[31] Beisken S, Earll M, Baxter C, Portwood D, Ament Z, Kende A, Hodgman C, Seymour G, Smith R, Fraser P et al (2014) Metabolic differences in ripening of *Solanum lycopersicum* “Ailsa Craig” and three monogenic mutants. Sci Data 1:140029. 10.1038/sdata.2014.29 25977786 10.1038/sdata.2014.29 PMC4322568

[32] Žuvela P, Skoczylas M, Jay Liu J, Ba̧czek T, Kaliszan R, Wong MW, Buszewski B (2019) Column characterization and selection systems in reversed-phase high-performance liquid chromatography. Chem Rev 119:3674–3729. 10.1021/acs.chemrev.8b00246 30604951 10.1021/acs.chemrev.8b00246

[33] Yang K, Swanson K, Jin W, Coley C, Eiden P, Gao H, Guzman-Perez A, Hopper T, Kelley B, Mathea M et al (2019) Analyzing learned molecular representations for property prediction. J Chem Inf Model 59:3370–3388. 10.1021/acs.jcim.9b00237 31361484 10.1021/acs.jcim.9b00237 PMC6727618

[34] Heid E, Greenman KP, Chung Y, Li S-C, Graff DE, Vermeire FH, Wu H, Green WH, McGill CJ (2023) Chemprop: a machine learning package for chemical property prediction. J Chem Inf Model 64:9–17. 10.1021/acs.jcim.3c01250 38147829 10.1021/acs.jcim.3c01250 PMC10777403

[35] Burges, C., Shaked, T., Renshaw, E., Lazier, A., Deeds, M., Hamilton, N., and Hullender, G. (2005). Learning to rank using gradient descent. Proceedings of the 22nd international conference on Machine learning. Association for Computing Machinery.

[36] Paszke, A., Gross, S., Massa, F., et al. PyTorch: An Imperative Style, High-Performance Deep Learning Library in Advances in Neural Information Processing Systems *32* (2019).

[37] Kingma, D. P. & Ba, J. Adam: a method for stochastic optimization 2015. arXiv: 1412.6980 .

[38] Lakshminarayanan, B., Pritzel, A., and Blundell, C. (2017). Simple and scalable predictive uncertainty estimation using deep ensembles. Proceedings of the 31st International Conference on Neural Information Processing Systems. Curran Associates Inc.

[39] Rodríguez-Pérez R, Trunzer M, Schneider N, Faller B, Gerebtzoff G (2022) Multispecies machine learning predictions of in vitro intrinsic clearance with uncertainty quantification analyses. Mol Pharm 20:383–394. 10.1021/acs.molpharmaceut.2c00680 36437712 10.1021/acs.molpharmaceut.2c00680

[40] Scalia G, Grambow CA, Pernici B, Li Y-P, Green WH (2020) Evaluating scalable uncertainty estimation methods for deep learning-based molecular property prediction. J Chem Inf Model 60:2697–2717. 10.1021/acs.jcim.9b00975 32243154 10.1021/acs.jcim.9b00975

[41] Van der Maaten L, Hinton G (2008) Visualizing data using t-SNE. J Mach Learn Res 9:2579–2605

[42] Tsugawa H, Nakabayashi R, Mori T, Yamada Y, Takahashi M, Rai A, Sugiyama R, Yamamoto H, Nakaya T, Yamazaki M et al (2019) A cheminformatics approach to characterize metabolomes in stable-isotope-labeled organisms. Nat Methods 16:295–298. 10.1038/s41592-019-0358-2 30923379 10.1038/s41592-019-0358-2

[43] Duhrkop K, Fleischauer M, Ludwig M, Aksenov AA, Melnik AV, Meusel M, Dorrestein PC, Rousu J, Bocker S (2019) SIRIUS 4: a rapid tool for turning tandem mass spectra into metabolite structure information. Nat Methods 16:299–302. 10.1038/s41592-019-0344-8 30886413 10.1038/s41592-019-0344-8

[44] Basic Concepts and the Control of Separation. (2009). In Introduction to Modern Liquid Chromatography, pp. 19-86. 10.1002/9780470508183.ch2

[45] Gritti F (2021) Perspective on the future approaches to predict retention in liquid chromatography. Anal Chem 93:5653–5664. 10.1021/acs.analchem.0c05078 33797872 10.1021/acs.analchem.0c05078

[46] Soriano-Meseguer S, Fuguet E, Port A, Rosés M (2019) Influence of the acid-base ionization of drugs in their retention in reversed-phase liquid chromatography. Anal Chim Acta 1078:200–211. 10.1016/j.aca.2019.05.063 31358220 10.1016/j.aca.2019.05.063

[47] Souihi A, Mohai MP, Palm E, Malm L, Kruve A (2022) MultiConditionRT: predicting liquid chromatography retention time for emerging contaminants for a wide range of eluent compositions and stationary phases. J Chromatogr A. 10.1016/j.chroma.2022.462867 10.1016/j.chroma.2022.462867 35139450

[48] Haidar Ahmad IA, Shchurik V, Nowak T, Mann BF, Regalado EL (2020) Introducing multifactorial peak crossover in analytical and preparative chromatography via computer-assisted modeling. Anal Chem 92:13443–13451. 10.1021/acs.analchem.0c02807 32786491 10.1021/acs.analchem.0c02807

[49] Haddad PR, Taraji M, Szücs R (2020) Prediction of analyte retention time in liquid chromatography. Anal Chem 93:228–256. 10.1021/acs.analchem.0c04190 33085452 10.1021/acs.analchem.0c04190

[50] Schymanski EL, Jeon J, Gulde R, Fenner K, Ruff M, Singer HP, Hollender J (2014) Identifying small molecules via high resolution mass spectrometry: communicating confidence. Environ Sci Technol 48:2097–2098. 10.1021/es5002105 24476540 10.1021/es5002105

