# Supplementary material for: ROASMI: accelerating small molecule identification by repurposing retention data
Source: J Cheminform. 2025 Feb 14;17:20. doi: 10.1186/s13321-025-00968-8 (PMC11829455; doi:10.1186/s13321-025-00968-8)
Supplement: Supplementary file 1 — Additional file 1 [file 13321_2025_968_MOESM1_ESM.docx]

Supplementary Figures


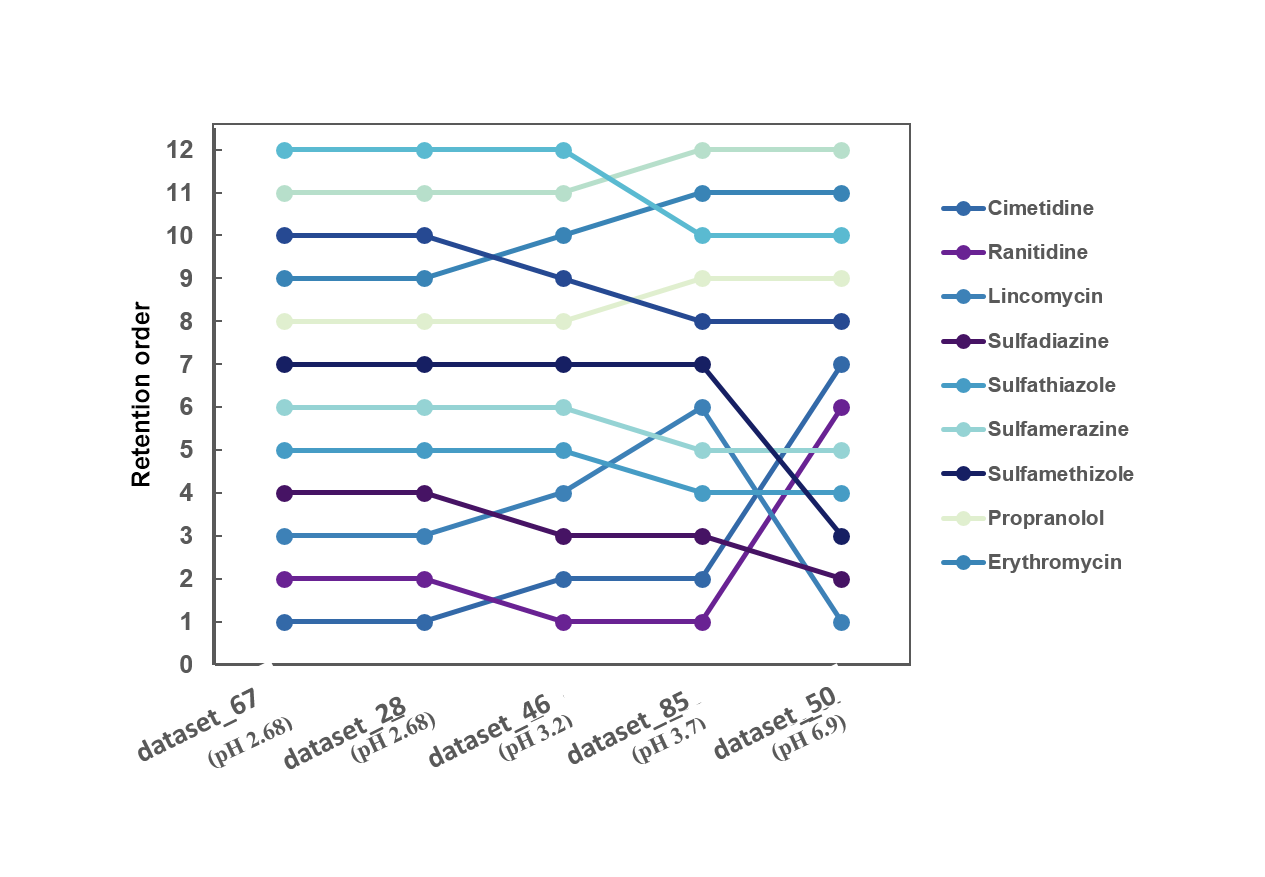


**Supplementary Fig. 1** Retention order of 12 shared compounds in five cross-laboratory datasets.


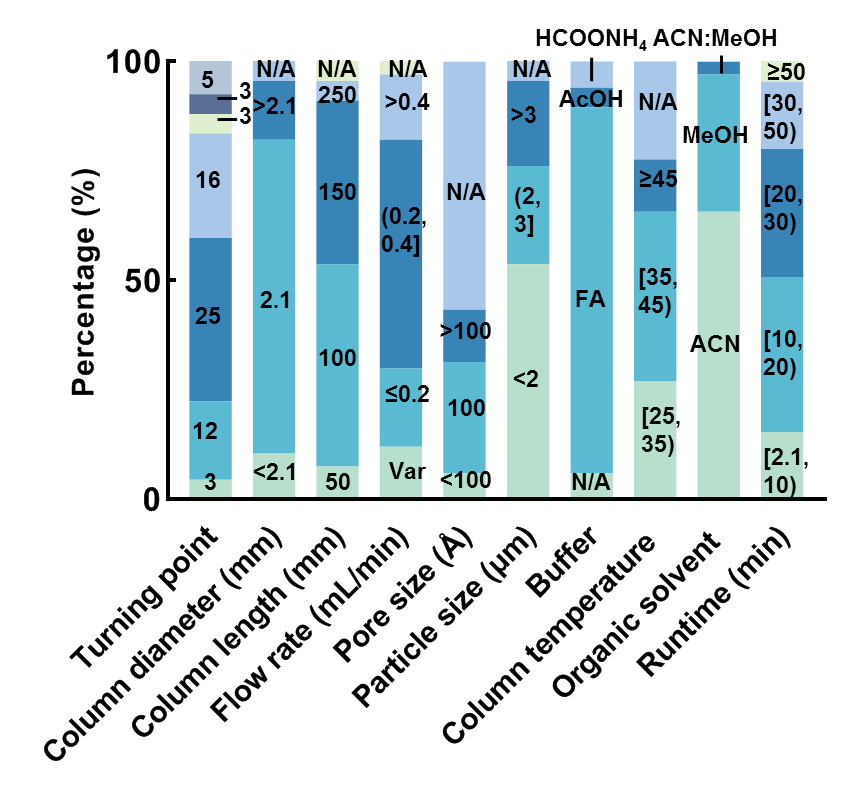


**Supplementary Fig. 2** The distribution of chromatographic conditions for 71 independent datasets. “Turning point” refers to the point in the gradient profile when the slope changes, and its number can reflect the degree of nonlinearity of the gradient to some extent.


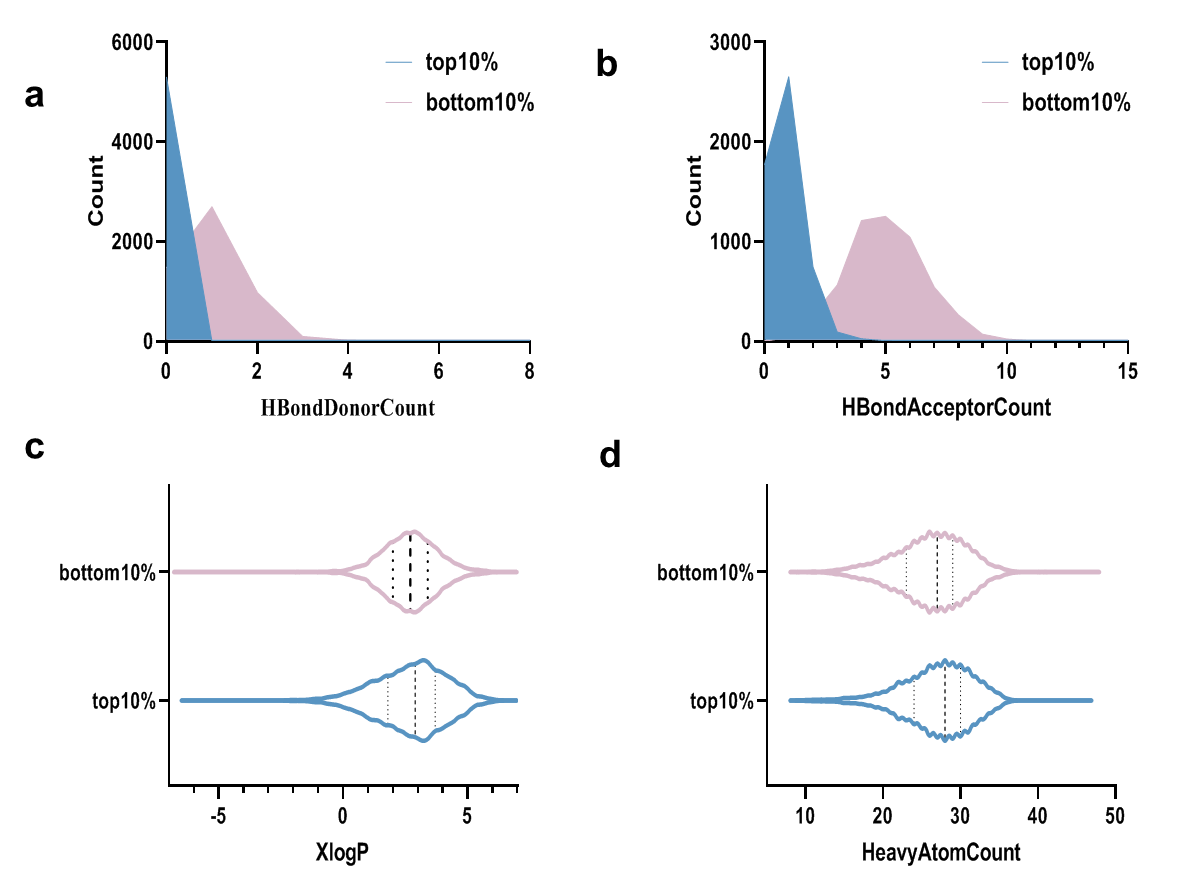


**Supplementary Fig. 3** Influence of the compound properties on the initial model performance. (**a-b**) Distribution of Hydrogen Bond Donor Count (a) and Hydrogen Bond Acceptor Count (b) between the top-performing (top 10%) and bottom-performing (bottom 10%) compound sets. (**c-d**) Comparison of XlogP (**c**) and Heavy Atom Count (**d**) between the top 10% and bottom 10% compound sets. The left and right hinges represent the first and third quartiles, respectively, while the center line represents the median and mean.

******Supplementary Fig. 4** The effect of the amount of reference data on the retraining outcome. As exemplified by an extended A10 experiment.

**Supplemental Tables**

Supplemental Table 1 ROASMI application on dataset_71 to distinguish isomers

| Isomers | Compound | PubChem  CID | RT (min) | Retention  score | Original ranking | New ranking |
| --- | --- | --- | --- | --- | --- | --- |
| Pair 1 | Delta-Hexanolactone | 13204 | 6.3 | -2.86 | no distinction | correct |
|  | Gamma-Caprolactone | 12756 | 7.5 | -2.73 |  |  |
| Pair 2 | Tyramine | 5610 | 2.9 | -5.68 | no distinction | wrong |
|  | 2-Hydroxyphenethylamine | 1000 | 3.8 | -6.05 |  |  |
| Pair 3 | Methylglutaric acid | 12284 | 5.9 | -4.98 | correct | correct |
|  | Monomethyl glutaric acid | 73917 | 6.9 | -4.90 |  |  |
| Pair 4 | 3-Methyladenine | 135398661 | 1.8 | -5.66 | correct | wrong |
|  | 6-Methyladenine | 67955 | 3.1 | -6.36 |  |  |
| Pair 5 | 2-Phenylglycine | 3866 | 2.1 | -5.00 | correct | wrong |
|  | Acetaminophen | 1983 | 4.9 | -5.26 |  |  |
| Pair 6 | 4-Hydroxy-3-methylbenzoic acid | 68138 | 9.5 | -3.65 | correct | correct |
|  | p-Anisic acid | 7478 | 11.6 | -3.05 |  |  |
| Pair 7 | L-Phenylalanine | 6140 | 4.4 | -4.07 | correct | correct |
|  | Benzocaine | 2337 | 11.6 | -1.52 |  |  |
| Pair 8 | Phenylephrine | 6041 | 2.7 | -6.84 | correct | correct |
|  | 3-Methoxytyramine | 1669 | 3.7 | -5.29 |  |  |
| Pair 9 | 12-Hydroxydodecanoic acid | 79034 | 16.9 | 3.65 | correct | correct |
|  | 3-Hydroxydodecanoic acid | 94216 | 18.8 | 10.46 |  |  |
| Pair 10 | Estriol | 5756 | 12.8 | -1.59 | no distinction | correct |
|  | Epiestriol | 68929 | 14.4 | -1.35 |  |  |
| Pair 11 | m-Aminobenzoic acid | 7419 | 3.9 | -4.33 | correct | correct |
|  | p-Aminobenzoic acid | 978 | 5 | -3.96 |  |  |
| Triplet 1 | Glutaric acid | 743 | 3.9 | -6.10 | correct | correct |
|  | Methylsuccinic acid | 10349 | 4.7 | -5.45 |  |  |
|  | Monoethyl malonic acid | 70615 | 5.5 | -4.96 |  |  |
| Triplet 2 | Homogentisic acid | 780 | 4.1 | -4.27 | no distinction | correct |
|  | Vanillic acid | 8468 | 7.8 | -3.57 |  |  |
|  | 5-Methoxysalicylic acid | 75787 | 10.5 | -2.75 |  |  |

Supplemental Table 2 ROASMI application on dataset_79 to distinguish isomers

| Isomers | Compound | PubChem_CID | RT (min) | Retention_score | Ranking |
| --- | --- | --- | --- | --- | --- |
| Pair 1 | 4-quinolinecarboxylic acid | 10243 | 1.91 | -3.32 | correct |
|  | 2-quinolinecarboxylic acid | 7124 | 3.21 | -2.22 |  |
| Pair 2 | n-(3-phenylpropionyl) glycine | 152323 | 2.34 | -3.60 | correct |
|  | n-acetyl-phenylalanine | 74839 | 3.99 | -3.24 |  |
| Pair 3 | 4-imidazoleacetic acid | 96215 | 0.82 | -6.71 | wrong |
|  | thymine | 1135 | 1.88 | -7.13 |  |
| Pair 4 | methylsuccinic acid | 10349 | 2.58 | -5.45 | correct |
|  | 3,5-dimethoxyphenol | 10383 | 4.13 | -1.99 |  |
| Pair 5 | 6-hydroxynicotinic acid | 72924 | 1.96 | -5.48 | correct |
|  | 4-nitrophenol | 980 | 4.18 | -2.24 |  |
| Pair 6 | pyrogallol | 1057 | 1.94 | -3.85 | wrong |
|  | 3-hydroxy-2-methyl-4-pyrone | 8369 | 2.77 | -5.23 |  |
| Pair 7 | 4-aminophenol | 403 | 0.83 | -5.00 | correct |
|  | 2-aminophenol | 5801 | 1.42 | -4.55 |  |
| Pair 8 | gallic acid | 370 | 1.88 | -4.35 | correct |
|  | 2,3,4-trihydroxybenzoic acid | 11874 | 2.59 | -3.93 |  |
| Pair 9 | noradrenaline | 439260 | 0.81 | -7.12 | correct |
|  | pyridoxine | 1054 | 1.54 | -5.92 |  |
| Pair 10 | suberic acid | 10457 | 4.06 | -4.71 | correct |
|  | diethyl succinate | 31249 | 4.61 | -2.70 |  |
| Pair 11 | synephrine | 7172 | 0.85 | -7.42 | correct |
|  | 3-methoxytyramine | 1669 | 1.99 | -5.29 |  |
| Pair 12 | p-coumaric acid | 637542 | 3.76 | -3.15 | correct |
|  | 3-hydroxycinnamic acid | 637541 | 4.38 | -2.88 |  |
| Pair 13 | 5-hydroxyindole | 16054 | 2.90 | -1.62 | correct |
|  | 3-hydroxyindole | 50591 | 3.85 | -1.47 |  |
| Pair 14 | 1,5,6,7-tetrahydro-4h-indol-4-one | 280229 | 2.95 | -2.79 | correct |
|  | n-benzylformamide | 80654 | 3.67 | -2.29 |  |
| Triplet 1 | 4-hydroxyproline | 440014 | 0.75 | -7.98 | correct |
|  | propionylglycine | 98681 | 1.70 | -7.26 |  |
|  | n-acetyl-alanine | 88064 | 1.77 | -6.48 |  |
| Triplet 2 | salicylic acid | 338 | 2.19 | -3.19 | wrong |
|  | 3,4-dihydroxybenzaldehyde | 8768 | 2.91 | -3.53 |  |
|  | 3-hydroxybenzoic acid | 7420 | 3.47 | -3.80 |  |
| Triplet 3 | homogentisic acid | 780 | 2.23 | -4.27 | correct |
|  | vanillic acid | 8468 | 3.33 | -3.57 |  |
|  | 5-methoxysalicylic acid | 75787 | 4.48 | -2.75 |  |
| Triplet 4 | trigonelline | 5570 | 0.82 | 1.23 | wrong |
|  | 4-aminobenzoic acid | 978 | 2.41 | -3.96 |  |
|  | methyl nicotinic acid | 7151 | 3.23 | -3.59 |  |
| Quadruplet 1 | 4-hydroxyphenyl acetic acid | 127 | 3.23 | -4.41 | correct |
|  | 2-hydroxyphenylacetic acid | 11970 | 3.60 | -4.05 |  |
|  | 2',4'-dihydroxyacetophenone | 6990 | 4.09 | -3.42 |  |
|  | 3-methylsalicylic acid | 6738 | 5.29 | -3.01 |  |
| Quadruplet 2 | hydroxyphenyllactic acid | 9378 | 2.87 | -4.10 | correct |
|  | homovanillic acid | 1738 | 3.42 | -4.08 |  |
|  | 2,6-dimethoxybenzoic acid | 15109 | 3.65 | -3.00 |  |
|  | methyl vanillate | 19844 | 4.36 | -2.25 |  |
| Quintuplet | 3-amino-4-hydroxybenzoic acid | 65083 | 1.25 | -4.79 | correct |
|  | 3-amino-5-hydroxybenzoic acid | 127115 | 1.32 | -4.68 |  |
|  | 3-hydroxyanthranilic acid | 86 | 2.57 | -4.13 |  |
|  | 4-aminosalicylic acid | 4649 | 2.69 | -3.83 |  |
|  | salicylhydroxamic acid | 66644 | 3.04 | -3.48 |  |

Supplemental Table 3 Re-annotation results for dataset_38

| Raw (as Ground Truth) | | | | Scenario 1 | | Scenario 2 | | Scenario 3 | |
| --- | --- | --- | --- | --- | --- | --- | --- | --- | --- |
| CHEBI  ID | Adduct | RT  (min) | m/z | ROASMI only | No. of  Cand. | MS-DIAL only | No. of  Cand. | Combo. | No. of  Cand. |
| 16941 | M-H | 1.64 | 217.0299 | n/a | | No MS/MS | Wrong formula | n/a | |
| 28853 | M-H | 1.66 | 245.0431 |  |  |  |  |  |  |
| 21363 | M-H | 1.90 | 182.0462 |  |  |  |  |  |  |
| 9538 | M-H | 1.97 | 226.9968 |  |  |  |  |  |  |
| 16426 | M-H | 2.09 | 219.0151 |  |  |  |  |  |  |
| 16108 | M-H | 9.30 | 168.9897 |  |  |  |  |  |  |
| 3159 | M-H | 13.47 | 309.1746 |  |  |  |  |  |  |
| 16934 | M-H | 1.51 | 113.033 |  |  |  |  |  |  |
| 28386 | M-H | 1.62 | 149.0637 |  |  |  |  |  |  |
| 17964 | M+H | 2.79 | 130.0861 |  |  |  |  |  |  |
| 30915 | M-H | 3.04 | 145.0145 |  |  | n/a |  |  |  |
| 91030 | M-H | 4.43 | 391.0319 |  |  |  |  |  |  |
| 30881 | M-H | 4.71 | 196.0227 |  |  |  |  |  |  |
| 27951 | M-H | 9.30 | 212.9794 |  |  |  |  |  |  |
| 17368 | M-H | 1.75 | 135.0301 |  |  |  |  |  |  |
| 2365 | M-H | 10.13 | 265.1484 |  |  |  |  |  |  |
| 48095 | M+Na | 1.57 | 203.0526 |  |  |  |  |  |  |
| 18237 | M-H | 1.62 | 146.0462 |  |  | No MS/MS | No formula |  |  |
| 27904 | M-H | 1.67 | 119.035 |  |  |  |  |  |  |
| 48093 | M-H | 1.69 | 165.0405 |  |  |  |  |  |  |
| 36124 | M-H | 1.92 | 191.0563 |  |  |  |  |  |  |
| 18183 | M-H | 4.72 | 128.0354 |  |  |  |  |  |  |
| 30314 | M-H | 2.28 | 205.0355 |  |  | n/a |  |  |  |
| 30845 | M-H | 4.46 | 111.0088 |  |  | n/a |  |  |  |
| 18012 | M-H | 2.46 | 115.0038 | 3 | 9 | 0 | 5 | 0 | 11 |
| 15741 | M-H | 5.35 | 117.0193 | 3 | 12 | 0 | 11 | 0 | 20 |
| 17196 | M-H | 1.48 | 131.0456 | 9 | 16 | 0 | 13 | 0 | 26 |
| 17191 | M+H | 5.59 | 132.102 | 4 | 36 | 0 | 37 | 0 | 66 |
| 6650 | M-H | 2.44 | 133.0142 | 5 | 9 | 0 | 10 | 0 | 18 |
| 30763 | M-H | 9.88 | 137.0246 | 1 | 12 | 0 | 24 | 0 | 33 |
| 28300 | M-H | 1.54 | 145.0618 | 11 | 22 | 0 | 18 | 0 | 38 |
| 32805 | M-H | 4.46 | 173.0093 | 2 | 9 | 0 | 7 | 0 | 13 |
| 29016 | M+H | 1.35 | 175.1188 | 1 | 2 | 0 | 7 | 0 | 8 |
| 48131 | M-H | 9.61 | 187.0977 | 3 | 12 | 0 | 43 | 0 | 54 |
| 27897 | M+H | 8.87 | 205.0959 | 6 | 21 | 0 | 37 | 0 | 55 |
| 17764 | M-H | 1.64 | 215.0331 | 0 | 1 | 0 | 4 | 0 | 4 |
| 16704 | M-H | 5.79 | 243.0626 | 2 | 5 | 0 | 11 | 0 | 15 |
| 16750 | M-H | 6.26 | 282.08 | 1 | 7 | 0 | 18 | 0 | 23 |
| 16335 | M+FA-H | 6.01 | 312.0952 | 0 | 12 | 0 | 19 | 0 | 29 |
| 17992 | M+FA-H | 1.91 | 387.1149 | 154 | 213 | 0 | 62 | 0 | 274 |
| 27570 | M-H | 1.43 | 154.0622 | 3 | 8 | 0 | 8 | 1 | 14 |
| 30769 | M-H | 4.47 | 191.0199 | 0 | 19 | 1 | 7 | 0 | 25 |
| 30887 | M-H | 2.53 | 191.02 | 9 | 19 | 1 | 9 | 1 | 27 |
| 3293 | M-H | 9.05 | 341.0887 | 1 | 10 | 3 | 50 | 2 | 58 |
| 86511 | M-H | 4.33 | 195.0449 | 2 | 3 | 3 | 15 | 5 | 15 |
| 17215 | M-H | 10.69 | 194.0826 | 10 | 20 | 4 | 55 | 9 | 73 |
| 6086 | M-H | 10.18 | 311.1691 | 8 | 11 | n/a | 3 | 11 | 14 |
| 3393 | M-H | 1.73 | 377.0863 | 1 | 2 | n/a | 30 | 12 | 31 |
| 25097 | M-H | 2.05 | 149.0458 | 17 | 77 | n/a | 15 | 21 | 90 |
| 16680 | M-H | 1.92 | 383.1188 | 1 | 3 | n/a | 29 | 24 | 31 |
| 17270 | M-H | 1.53 | 171.0064 | 0 | 4 | No MS/MS | n/a | 0 | 4 |
| 17335 | M-H | 9.11 | 325.0937 | 0 | 20 |  |  | 0 | 20 |
| 30794 | M-H | 2.64 | 103.0037 | 1 | 4 |  |  | 1 | 4 |
| 1113 | M-H | 1.68 | 113.0246 | 1 | 12 |  |  | 1 | 12 |
| 17053 | M-H | 1.53 | 132.0305 | 1 | 5 |  |  | 1 | 5 |
| 15676 | M-H | 1.76 | 157.0368 | 1 | 4 |  |  | 1 | 4 |
| 33508 | M-H | 1.92 | 105.0194 | 2 | 3 |  |  | 2 | 3 |
| 32816 | M-H | 4.60 | 87.00869 | 2 | 7 |  |  | 2 | 7 |
| 36090 | M-H | 8.99 | 163.0404 | 2 | 23 |  |  | 2 | 23 |
| 33809 | M-H | 1.58 | 369.0681 | 2 | 6 |  |  | 2 | 6 |
| 17521 | M-H | 9.11 | 191.0563 | 2 | 10 |  |  | 2 | 10 |
| 16112 | M+H | 9.06 | 355.1032 | 2 | 20 |  |  | 2 | 20 |
| 17822 | M-H | 1.47 | 104.0353 | 3 | 8 |  |  | 3 | 8 |
| 17081 | M-H | 1.67 | 143.0352 | 3 | 26 |  |  | 3 | 26 |
| 16308 | M-H | 1.81 | 421.0764 | 3 | 22 |  |  | 3 | 22 |
| 16016 | M-H | 3.02 | 89.0242 | 3 | 13 |  |  | 3 | 13 |
| 30831 | M-H | 1.68 | 101.0244 | 4 | 14 |  |  | 4 | 14 |
| 16217 | M-H | 2.02 | 177.0408 | 4 | 35 |  |  | 4 | 35 |
| 16292 | M-H | 4.47 | 147.0299 | 5 | 28 |  |  | 5 | 28 |
| 17750 | M+H | 1.57 | 118.0862 | 6 | 28 |  |  | 6 | 28 |
| 16977 | M-H | 1.44 | 88.04027 | 6 | 19 |  |  | 6 | 19 |
| 16885 | M-H | 1.88 | 503.1623 | 6 | 469 |  |  | 6 | 469 |
| 17369 | M+H | 1.48 | 261.0376 | 49 | 68 |  |  | 49 | 68 |

‘n/a’ indicates no follow-up analysis, ‘combo.’ denotes the combined use of both MS-DIAL and ROASMI.
